# Changes in Host Cytokine Patterns of TB Patients with Different Bacterial Loads Detected Using ^16^S rRNA Analysis

**DOI:** 10.1371/journal.pone.0168272

**Published:** 2016-12-16

**Authors:** Rhiannon Heslop, Adama L. Bojang, Sheikh Jarju, Joseph Mendy, Sarah Mulwa, Ousman Secka, Francis S. Mendy, Olumuyiwa Owolabi, Beate Kampmann, Jayne S. Sutherland

**Affiliations:** 1 Vaccines and Immunity Theme, Medical Research Council (MRC) Unit, Banjul, The Gambia; 2 The University of Manchester, Oxford Rd, Manchester, United Kingdom; Fundació Institut d’Investigació en Ciències de la Salut Germans Trias i Pujol, Universitat Autònoma de Barcelona, SPAIN

## Abstract

**Background:**

Tuberculosis (TB) has overtaken HIV as the biggest infectious disease killer, with the majority of deaths occurring in sub-Saharan Africa. However it is unknown how differences in bacterial load alter host immune profiles in the sputum and blood of TB patients.

**Methods:**

^16^S ribosomal RNA analysis was used to determine bacterial load in sputum samples obtained from 173 patients with active TB (57 pre-treatment and 116 post-treatment). Host analyte concentrations in sputum and *Mycobacterium tuberculosis* (Mtb) antigen stimulated whole blood assay supernatants were analysed using multiplex cytokine arrays.

**Results:**

Multiple logistic regression adjusting for age, sex and HIV status showed highly significant correlation of bacterial load with IL1β, IL2, IL1RA, IL4, IL6, IL8, IL9, IL15, IL17, EOTAX, FGF, IFN-γ, GCSF, MCP1, M1P1α, M1P1β, PDGF, TNFα, VEGF in sputum. With increasing time on treatment, FGF levels in sputum displayed the most significant inverse correlation with reduction in bacterial load.

**Conclusions:**

We show that differences in bacterial load correlates with changes in several host biomarkers. These findings have implications for development of tests for TB diagnosis and treatment response.

## Introduction

Despite recent efforts, tuberculosis (TB) persists as a global health problem with an estimated 10.4 million new cases and 1.8 million deaths in 2015 [1]. TB is caused by inhalation of *Mycobacterium tuberculosis* (Mtb), a gram positive, acid-fast bacillus (AFB) [2]. Two major roadblocks in combating TB are the limitations of current diagnostic tests and difficulties in assessing the early treatment response. We hypothesise this is due in part to the large variability in Mtb bacterial load in individual patients.

The molecular bacterial load (MBL) assay amplifies the ^16^S ribosomal RNA of Mtb, which degrades much faster than DNA and therefore indicates the level of viable bacteria. It allows the fast and accurate quantification of bacterial burden and allows monitoring of patient response within the first three days of treatment [3].

Patients with multiple respiratory symptoms are likely to have higher bacterial loads, which are associated with poorer prognosis [4] and more extensive transmission of active TB [5]. Additionally, it has been shown that patients with higher colony forming units (CFUs) in their sputum are more likely to have cavitary disease [6]. Thus, determining the bacterial load would be beneficial for optimal patient management.

The identification of surrogate markers for bacterial load may help to predict treatment outcome, treatment response and risk of reactivation of TB similar to the use of viral load/CD4 count for determining disease severity and response to anti-retroviral therapy in HIV infected subjects [7]. Changes in host immune profiles in relation to bacterial load have been crudely studied previously using smear grade including differences in antibody profiles [8] and polyfunctional T cell profiles [9]. However, correlation of host markers with specific, quantifiable bacterial loads has not been performed to date.

We have previously shown that *ex vivo* host factors in sputum can accurately distinguish between TB and other respiratory diseases (ORD) [10] with levels significantly reducing as early as 2 weeks post treatment initiation (Sutherland et al, unpublished). Thus we hypothesised that these surrogate markers in sputum could be used to distinguish different bacterial levels at diagnosis and for treatment monitoring.

The aim of this study was to determine how differences in quantifiable bacterial load relate to differences in host immune profiles in sputum and blood before and after treatment initiation. Since higher bacterial burden has been shown to be an important risk factor for treatment failure and relapse, our findings have implications for patient management including diagnosis, prognosis and treatment monitoring.

## Methods

### Ethics statement

This work was approved by the MRC/Gambian government joint ethics committee. Written informed consent was provided by all study participants.

### Subjects and samples

173 HIV negative adult patients with smear-positive TB were recruited. Sputum was collected, digested using Sputolysin (Merck, USA) and centrifuged at 1500rpm. The supernatant was removed and stored for host cytokine/chemokine analysis at -20^°^C and the bacterial pellet was resuspended in Trizol (ThermoFisher Scientific, USA) and stored at -80^°^C until analysis. All samples were analysed by AFB-smear microscopy and GeneXpert MTB-RIF. Heparinised blood was collected from 86 subjects and stimulated overnight with Mtb antigens.

### Preparation of Mtb Standards for the MBL Assay

Five hundred microliters of wild-type Mtb (H37Rv) stock and 800μl of mycobacteria growth indicator tube (MGIT) growth supplement were added to a MGIT tube (Becton Dickinson, USA) and incubated in a BACTEC MGIT 960 (Becton Dickinson, USA) machine for five days. Viability was confirmed via a fluorescent reaction in the MGIT tube. The tube was then mixed by hand, and 500μl was inoculated into 20μl 7H9-Tween-20 and incubated at 37°C. Optical density (OD) was measured using a spectrophotometer every 2 days to assess the growth of the bacteria in conjunction with the McFarland scale. Once an OD of 2.2 was reached 1ml aliquots of the suspension were frozen at -80°C in Trizol. To confirm the top standard concentration, 10-fold serial dilutions of 10^−1^ to 10^−5^ were performed with 7H9 media on one aliquot. Three 20 μl drops were plated onto 7H11 agar and incubated at 37°C for three weeks and colony forming units (CFU) were counted.

### Extraction of RNA

Before extraction, 2 μl of 560 RNA Internal Control RNA (Bioline, UK) was spiked into 1ml sputum samples in Trizol. Two hundred microliters of chloroform was then added to each tube, samples were mixed vigorously and incubated at room temperature (RT) for 10 min. Samples were then centrifuged at 13,000 rpm for 15 mins and the upper aqueous phase was transferred to fresh tubes. An equal volume of 70% ethanol (approx. 600 μl) was added to each tube and mixed vigorously. The sample solutions were then transferred to RNeasy MiniElute Spin Columns (Qiagen, Netherlands) and RNA purified according to Qiagen protocol. For the standards, RNA was extracted and ten-fold serial dilutions performed using nuclease free water (Qiagen, The Netherlands).

### Molecular Bacterial Load Assay

Levels of ^16^S RNA and internal control (IC) were quantified using reverse transcription polymerase chain reaction (RT-PCR). To detect ^16^S RNA, a master mix containing 12.5 μl Quantitect Master Mix, 6.65 μl of nuclease free water, 0.25 μl reverse transcriptase, 0.3 μl of ^16^S-ROX (Rox-AGGACCACGGGATGCATGTCTTGT-BHQ2) (all supplied by Qiagen, Netherlands) per reaction was prepared. A master mix containing 12.5 μl Quantitect Master Mix, 5.05 μl of nuclease free water, 0.25 μl reverse transcriptase, 1.2 μl 50nM MgCl^2+^ (Qiagen, The Netherlands) and 1 μl VIC labelled 560 Control Mix (Bioline, UK) per reaction was prepared to detect the IC. 5μl of RNA standards, samples or H_2_0 were added to the 96 well plate in triplicates; 20 μl of ^16^S-ROX mastermix was added to two wells, 20 μl of IC mastermix was added to the third. The plate was briefly vortexed, then centrifuged at 10,000 rpm for 30s before being placed in 7500 Real-Time PCR System (Applied Biosystems, USA). The cycling parameters were set to 50°C for 30 mins, 95°C for 10 mins, then 45 cycles of 95°C for 15 secs and 60°C for 1 min. Analysis was performed on ABI 7500 software (version 2.3, Affymetrix, USA).

### Multiplex Cytokine Arrays

Sputum and Mtb-stimulated whole blood assay (WBA) supernatants were analysed using Bio-Plex Pro Human 27-Plex Kits (Bio-Rad, Belgium). 450ul of heparinised whole blood was stimulated with Purified protein derivative (PPD; SSI, Denmark) or ESAT-6 and CFP-10 (EC; kindly provided by Prof. Tom Ottenhoff, LUMC, The Netherlands; both at final concentration of 10μg/ml). Unstimulated (NIL) whole blood was used to determine background cytokine levels. Assays were conducted as previously described [10]. Analytes included IL-1β, IL-1ra, IL-2, IL-4, IL-5, IL-6, IL-7, IL-8, IL-9, IL-10, IL12p70, IL-13, IL-15, IL-17A, eotaxin, FGF Basic, G-CSF, GM-CSF, IFN-γ, IP-1O, MCP-1, MIP-1α, MIP-1β, PDGF-BB, RANTES, TNF-α and VEGF. Plates were read using a Magpix Multiplex Reader (BioRad, Belgium). Background values from the unstimulated wells were subtracted from EC and PPD values.

### Statistics

Spearman’s rank correlation was used to compare ^16^S RNA measurements with other measurements of bacterial load (smear microscopy, CXR, GeneXpert). A Mann-Whitney U test was used to compare pre and post-treatment MBL results. A Spearman’s Rank correlation matrix was used to determine if a correlation existed between mean bacterial load and cytokine profiles in sputum and WBA supernatants. From the associations observed a multiple logistic regression model was used to adjust for time on treatment, age, sputum smear grade, HIV status, gender and CXR severity. Statistical analyses were performed with Stata v. 14 (StataCorp, USA) or GraphPad Prism v.6.0 (Software MacKiev, USA). Adjustment for multiple comparisons was performed using a False Discovery Rate with p-values ≤0.035 considered significant.

## Results

### Patient demographics

Of 173 patients analysed, 24% were female and 75% male (Table 1) with a median[interquartile range (IQR)] age of 32.7[25.1–40.3] years. 80.3% of subjects were HIV negative with 4.6% confirmed positive and 15% unknown. The majority of subjects had sputum smear grade of 3+ (44.5%) and CXR score of 2 (moderate infiltration; 55.5%) (Table 1).

**Table 1:** Patient demographics.

**Characteristic**	**n(%)**
**Sex**	
Female	42 (24.3)
Male	129 (74.6)
Missing	2 (1.2)
**Chest X-ray**	
Zero	1 (0.6)
One	35 (20.2)
Two	96 (55.5)
Three	11 (6.4)
Missing	30 (17.3)
**HIV status**	
Negative	139 (80.3)
Positive	8 (4.6)
Missing	26 (15)
**Sputum smear grade**	
Zero	8 (4.6)
One Plus	33 (19.1)
Two Plus	53 (30.6)
Three Plus	77 (44.5)
Missing	2 (1.2)
**Age (Mean (SD))**	32.7 (12.2)
**Days on tx (Mean (SD))**	12.6 (20.2)

HIV, Human Immunodeficiency Virus; SD, standard deviation; tx, treatment

### Quantifying bacterial load in sputum using the MBL assay

**Fig 1:**
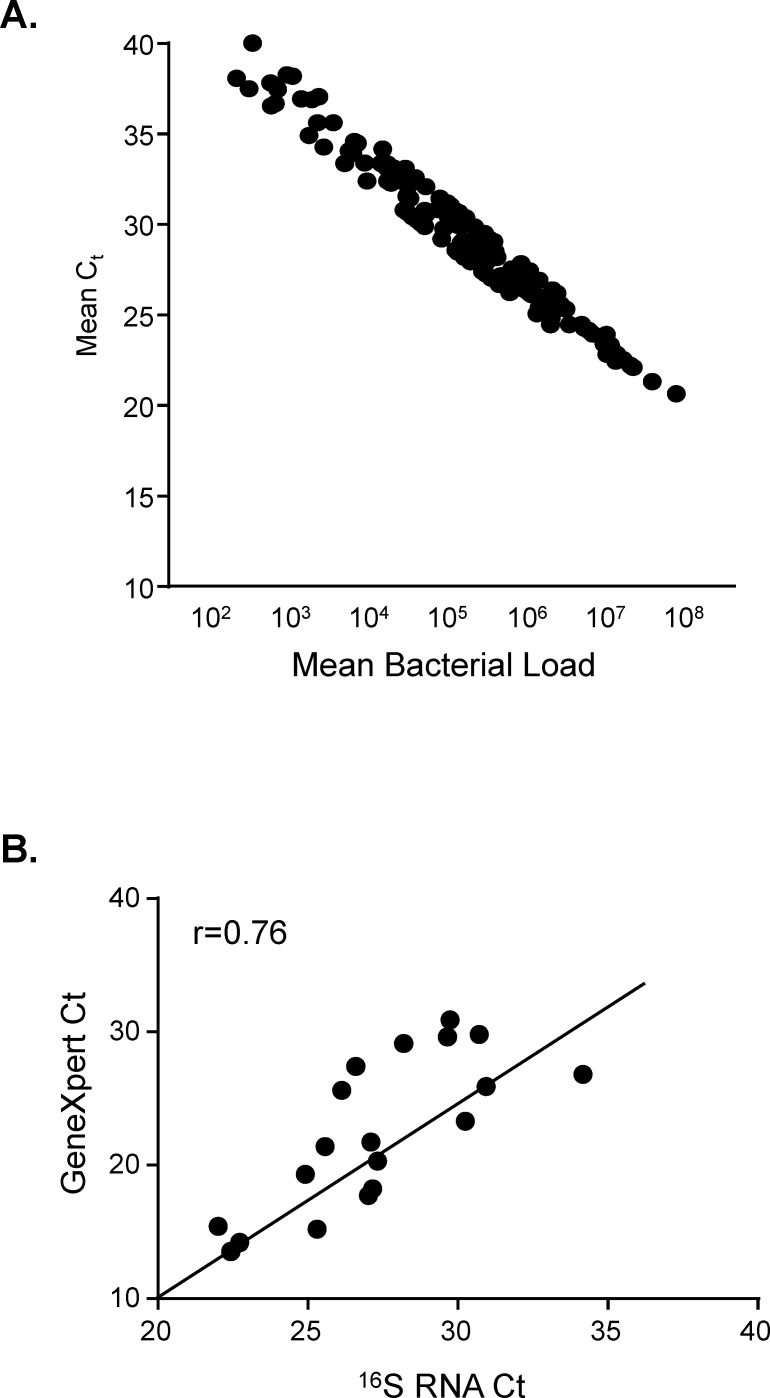
Correlation of bacterial load with cycle threshold values. RT-PCR was performed in duplicate on RNA extracted from sputum samples of TB patients. **A:** Log_10_ bacterial load versus Ct values of Mtb present in sputum pellets. **B:** Correlation of Ct values from GeneXpert and ^16^S RNA analysis. Data were analysed using Spearman rank correlation.

The mean Cycle threshold (Ct) value was determined from duplicate wells (the average CV for all samples was 2.1%) and construction of a standard curve allowed us to quantify the bacterial load. The upper and lower limits of detection using serially diluted standards were 10^8^ and 10^2^ cfu/ml respectively (Fig 1). Samples that were reported ‘undetermined’ by the software were assigned a Ct of 40 and a corresponding bacterial load of 100 cfu/ml for the purpose of statistical analysis. No influence of mycobacterial strain was observed on MBL assay results similar to our previous findings with GeneXpert [13]. The Ct values from the MBL assay and GeneXpert probe B showed strong correlation (r^2^ = 0.76, p = 0.0002; Fig 1B) at recruitment.

### Changes in Bacterial Load Pre- and Post-treatment

**Fig 2:**
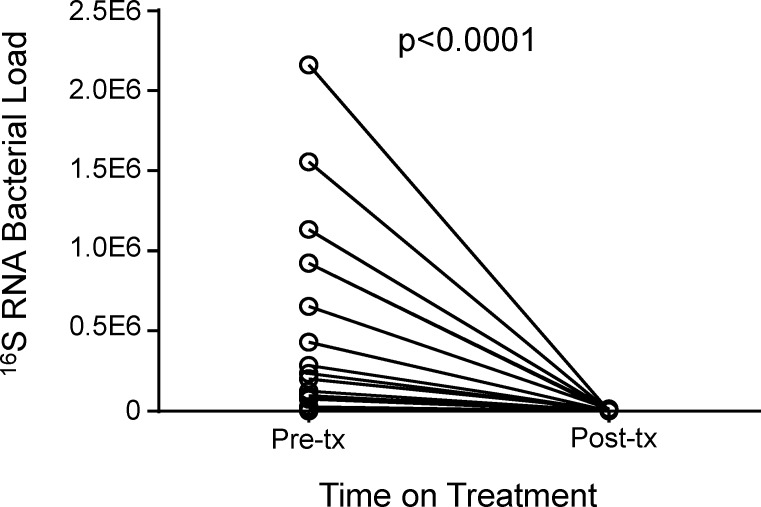
Mtb bacterial load pre- and post-treatment initiation. Sputum samples from patients pre and post treatment (n = 27) were analysed using the MBL assay. Line indicates median. Data were analysed using Wilcoxon matched pairs test.

The MBL assay was used to compare the bacterial loads of patient’s pre- and post-treatment initiation using Wilcoxon matched pairs analysis. The median bacterial load of patients pre-treatment was significantly higher than following treatment initiation (median [IQR] = 88107[19169–652533] copies pre-tx compared to 0[0–0] copies post-tx; p<0.0001; Fig 2). By 6 months, all subjects had 0 viable bacilli present in their sputum.

### Correlation of bacterial loads and host cytokine profiles

**Fig 3:**
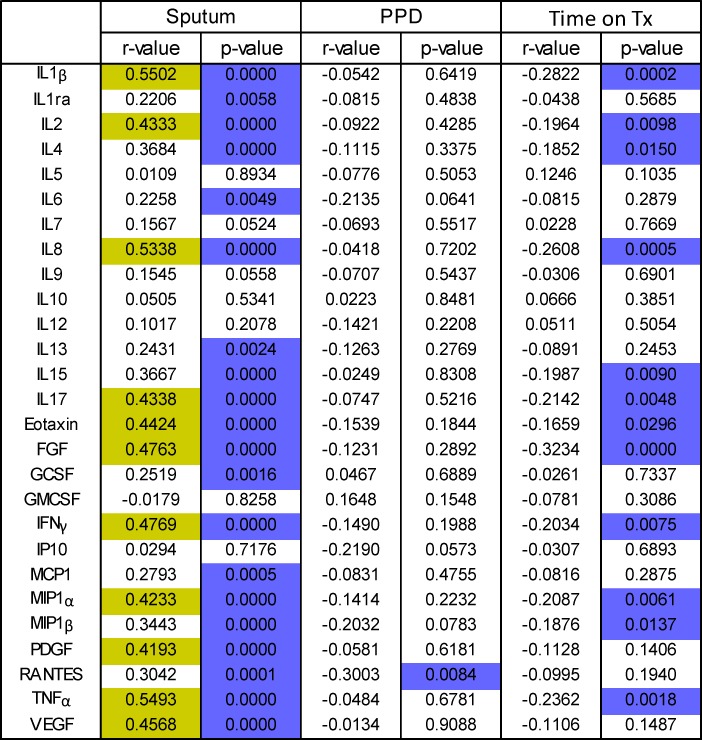
Correlation of MBL and host cytokines in blood and sputum. Bacterial load was analysed for each sputum sample and correlated with host biomarkers (pg/ml) in the same sputum sample and also in Mtb-stimulated and unstimulated blood using a 27-plex cytokine assay. R-values >0.4 are highlighted in yellow and significant p-values in purple. Data were analysed using a Spearman Rank Correlation. Correlation of Time-on treatment with host biomarkers in sputum was also performed.

A Spearman’s Rank correlation matrix was created to determine if there were significant correlations between bacterial load and cytokine profiles of WBA and sputum supernatants (Fig 3). There was no correlation with bacterial load at recruitment with the level of any cytokine from unstimulated (NIL) or EC stimulated whole blood. However there was a statistically significant inverse correlation between bacterial load and RANTES levels following PPD stimulation (r^2^ = -0.3, p = 0.0084). In digested sputum samples, the bacterial load correlated strongly (r>0.4) with levels of IL-1β (p< 0.0001), IL-2 (p<0.0001), IL-8 (p<0.0001), IL-17a (p<0.0001), Eotaxin (p<0.0001), FGF (p<0.0001), IFN-γ (p<0.0001), MIP-1α (p<0.0001), PDGF (p<0.0001), TNFα (p<0.0001) and VEGF (p<0.0001) (Fig 3). Bacterial load also correlated weakly (r<0.4) but significantly with IL-1ra (p = 0.0058), IL-4 (p<0.0001), IL-6 (p = 0.0049), IL-13 (p = 0.0024), IL-15 (p<0.0001), G-CSF (p = 0.0016), MCP-1 (p = 0.0005), MIP-1β (p<0.0001) and RANTES (p = 0.0001) (Fig 3).

**Fig 4:**
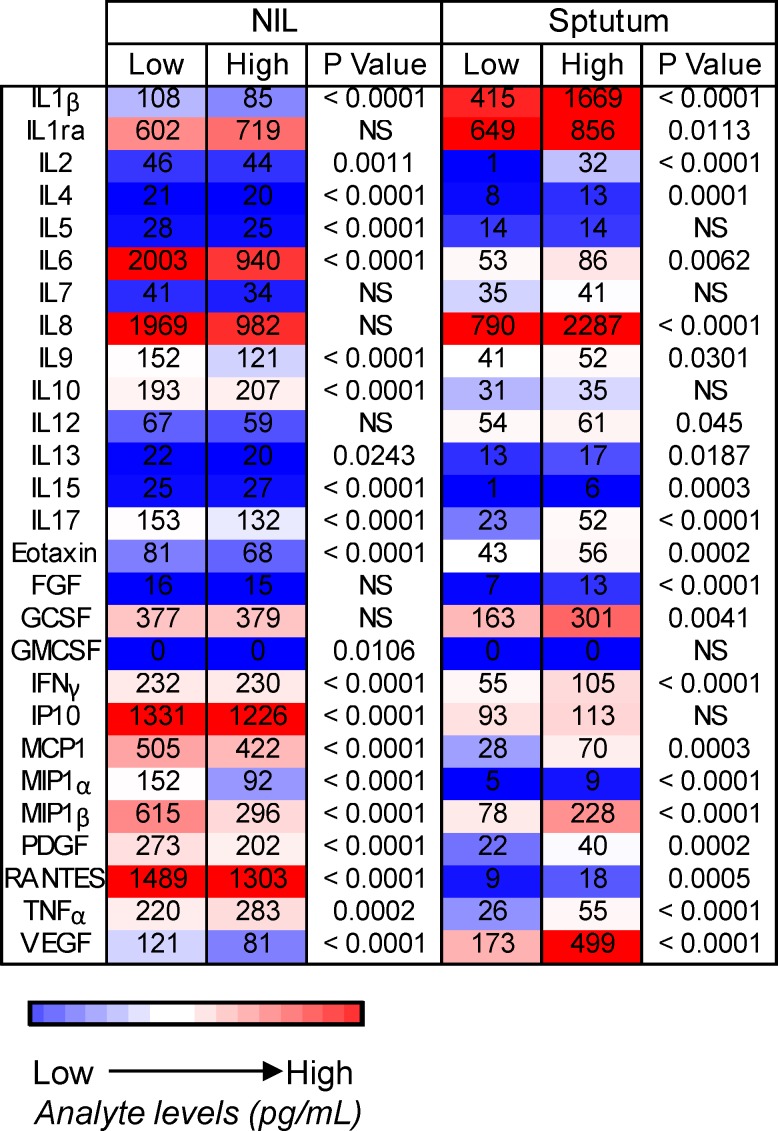
Heatmap of cytokine profiles in patients with low and high bacterial loads. Cytokine levels (pg/ml) for unstimulated (NIL) and sputum supernatants were analysed using a 27-plex cytokine array. Subjects were grouped into those with low bacterial load (below the median of 48059 copies/ml) and high bacterial load (above median). Data were analysed using a Mann-Whitney U-test. NS = not significant. Colour indicates low (blue) to high (red) cytokine levels.

When sputum analytes were correlated with days on TB therapy we found a significant inverse correlation with levels of IL1β, IL2, IL4, IL8, IL15, IL17, Eotaxin, FGF, IFN-γ, MIP-1α, MIP-1β and TNF-α (Fig 4B). The strongest inverse correlation was with FGF (r = -0.3234; Fig 3). Following multiple logistic regression adjusting for time on therapy, age, sex, HIV status, smear grade and CXR score, we found a significant correlation between bacterial load and levels of IL1β, IL2, IL4, IL8, IL17, Eotaxin, FGF, IFN-γ, MIP1α, MIP1β, PDGF and TNFα (all p<0.0001).

### Comparison of cytokine profiles in patients with high and low bacterial loads

The samples were then grouped into high and low bacterial load, depending on whether they were above or below the median (48059 copies/ml) bacterial load respectively. There were no differences in any analyte following EC and PPD stimulation (data not shown). However, the majority of analytes showed significant differences in the *ex vivo* samples (unstimulated blood and sputum). Median levels of IL-15, MIP-1α and TNF-α both increased significantly in NIL and sputum samples in subjects with high versus low bacterial load (Fig 4). Conversely, IL-2, IL4, IL6, IL9, IL13, IL17, Eotaxin, IFN-γ, MCP-1, MIP-1b, PDGF, RANTES and VEGF all decreased significantly in NIL but increased significantly in sputum samples with high bacterial load (Fig 4). Additionally, IL1ra, IL8, FGF and GCSF all increased significantly in sputum but showed no difference in NIL samples in subjects with high versus low bacterial load while IL4, IL10, GMCSF and IP10 all showed differences in NIL but not sputum samples (Fig 4).

## Discussion

The aim of this study was to determine changes in Mtb load in patients before and after initiation of TB therapy using ^16^S rRNA analysis and correlate this with host immune biomarkers in sputum and blood. We were able to show a significant correlation of quantifiable, viable bacterial load with multiple host factors in sputum and unstimulated whole blood.

Bacterial load determined using ^16^S RNA analysis showed a highly significant correlation with GeneXpert MTB-RIF Ct values before treatment initiation. However, the MBL assay has two major advantages over the GeneXpert: firstly it will only detect viable bacteria and can therefore be used to monitor treatment response and secondly, it can provide quantitative analysis of bacterial load, which allows for more accurate diagnosis and treatment monitoring. In addition, we have shown MBL to correlate with host biosignatures before and after treatment initiation, which could be used for generation of surrogate markers of disease severity and treatment response.

A significant decline in bacterial load was found as early as 1 week after treatment initiation. Furthermore, we were able to show a significant inverse correlation with time on treatment and the level of several host factors in the same sputum sample used for the MBL assay. This included IFN-γ, TNF-α, IL2, IL4, IL15, IL17, IL1β, MIP1α/β, FGF and Eotaxin suggesting a general reduction in the pro-inflammatory response. The most highly correlated analyte with treatment response was FGF, which we have previously shown to accurately distinguish between TB and other respiratory diseases (ORD) in sputum samples [10].

Many studies have assessed the difference between immune profiles of latent and active TB disease [ie 11], and the changes in cytokines during bacterial clearance (determined by smear microscopy or sputum culture) ie [12], but there is a paucity of information relating to differences in the host immune profiles depending on the severity of active disease (or bacterial load). Most studies find no or weak correlation of peripheral blood Mtb responses to mycobacterial load determined using smear or GeneXpert [13]. Indeed, in our study, when analysing antigen-stimulated whole blood, the only significant correlation (albeit relatively weak) was seen with the level of RANTES following PPD-stimulation with no differences seen following EC stimulation or in the unstimulated samples. However, in sputum we found 20/27 analytes showed significant positive correlation between bacterial and host levels in the same sputum sample. Of the cytokines we found significant correlations with, we have previously shown that IL-1β, IL-4, IL-7, IL-8, IL-13, IL-17, G-CSF, MCP-1 and TNFα are present in higher concentrations in *ex vivo* sputum than in serum or Mtb-stimulated blood [10]. This suggests that there are more pronounced changes in the immune response in the lungs during Mtb infection, which are more readily and accurately measured in sputum samples from the site of infection, rather than monitoring the concentrations of cytokines in the blood.

When we analysed samples based on high and low bacterial load, there were no significant differences in the cytokine values of whole blood stimulated with EC or PPD. However, there was a significant shift of the immune profile in unstimulated blood, with 17 of the 27 cytokines analysed (IL-1β, IL-2, IL-4, IL-5, IL-6, IL-9, IL-13, IL-17, Eotaxin, IFN-γ, IP-10, MCP-1, MIP-1α, MIP-1β, PDGF, RANTES, VEGF) being significantly lower in patients with higher bacterial load and levels of IL10, IL15 and TNF-α being higher. Unstimulated blood levels of IFN-γ have been found to be lower in active compared to latently infected people, whereas IL-4, IP-10, and VEGF have all been higher [13]. TNF-α has also been shown to be protective in latently infected subjects but associated with lung pathology and cavitary disease in subjects with higher levels in active TB disease [14]. Many of the cytokine levels that decreased in unstimulated blood increased in the sputum, possibly due to sequestration of activated cells to the site of infection as shown previously by our group [15]. The most significant increases were in IL-1β, IL-2, IL-8, IL-17, FGF, IFN-γ, MIP-1α, MIP-1β, TNF-α and VEGF. These factors all contribute to prolonged inflammation and tissue damage during disease. Following multiple logistic regression adjusting for time on therapy, age, sex, HIV status, smear grade and CXR score, we found a significant correlation between bacterial load and levels of IL1β, IL2, IL4, IL8, IL17, Eotaxin, FGF, IFN-γ, MIP1α, MIP1β, PDGF and TNFα.

In conclusion, we have demonstrated the clinical utility of the MBL assay for diagnosis and treatment response in pulmonary TB patients. Our data highlight the importance of analysing samples from the site of infection and provide novel correlates of bacterial load that could be developed further as a point-of-care assay following validation in different subject groups. Our findings have implications for patient management at diagnosis and treatment response monitoring.
